# Strain Tuning the Occupation of Candidate Topological Weyl States in W‐Doped MoTe_2_

**DOI:** 10.1002/advs.76064

**Published:** 2026-06-12

**Authors:** Amon Lanz, Olena Tkach, Yaryna Lytvynenko, Florian Diekmann, Harshit Agarwal, Sofia Michaela Souliou, Mehdi Frachet, Michael Merz, Sergii V. Chernov, Andrei Gloskovskii, Volkmar Koller, Dmytro Kutnyakhov, Moritz Hoesch, Christoph Schlueter, Philipp Rüssmann, Yuriy Mokrousov, Matthieu Le Tacon, Kai Rossnagel, Jure Demsar, Gerd Schönhense, Hans‐Joachim Elmers, Olena Fedchenko

**Affiliations:** ^1^ Institut für Physik Johannes Gutenberg‐Universität Mainz Germany; ^2^ Sumy State University Sumy Ukraine; ^3^ Institute of Magnetism of the NAS and MES of Ukraine Kyiv Ukraine; ^4^ Institut für Experimentelle und Angewandte Physik Christian‐Albrechts‐Universität zu Kiel Kiel Germany; ^5^ Institute for Quantum Materials and Technologies Karlsruhe Institute of Technology Karlsruhe Germany; ^6^ Institut Néel CNRS/UGA UPR2940 Grenoble France; ^7^ Karlsruhe Nano Micro Facility (KNMFi) Karlsruhe Institute of Technology Karlsruhe Germany; ^8^ Deutsches Elektronen‐Synchrotron DESY Hamburg Germany; ^9^ Peter Grünberg Institut and Institute for Advanced Simulation Forschungszentrum Jülich and JARA Jülich Germany; ^10^ Ruprecht Haensel Laboratory Deutsches Elektronen‐Synchrotron DESY Hamburg Germany; ^11^ Physikalisches Institut Goethe Universität Frankfurt Frankfurt am Main Germany

**Keywords:** condensed matter physics, conductivity, electrical resistivity and conductivity, electronic states, electronic structure, materials science, metastability, orthorhombic crystal system, photoemission spectroscopy, scattering

## Abstract

Strain‐induced modifications of the electronic structure in $T_{d}$‐${\mathrm{Mo}}_{0.91}W_{0.09}{\mathrm{Te}}_{2}$ were studied using hard X‐ray angle‐resolved photoemission spectroscopy (HARPES). Partial substitution of Mo with W stabilizes the metastable low‐temperature orthorhombic $T_{d}$ phase of ${\mathrm{MoTe}}_{2}$. Samples were first characterized by angle‐resolved photoemission spectroscopy experiments with soft X‐rays and higher resolution, showing good agreement with ab initio calculations of electronic states related to topology. The modification of the bulk electronic structure due to the applied strain was assessed using operando HARPES. Applying tensile strain along the a‐axis with amplitudes up to 0.34% result in changes in the bulk electronic structure as predicted by previous ab‐initio calculations. In particular, the results demonstrate a strain‐driven depletion of electronic states with heavy masses and high scattering rates, leading to an increase in conductivity. In contrast, tensile strain along the b‐axis results in a significant increase in resistivity, which we confirmed by transport studies.

## Graphical abstract

The present study investigates strain‐induced modifications of the electronic structure in the Weyl semimetal $T_{d}$‐${\mathrm{Mo}}_{0.91}W_{0.09}{\mathrm{Te}}_{2}$ using hard X‐ray angle‐resolved photoemission spectroscopy. Tensile strain along the a‐axis has been demonstrated to induce an increase in the Fermi wave vector in reciprocal space and a depletion of electronic states with heavy masses.

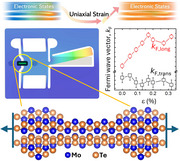

## 1 Introduction

The two‐dimensional electronic structure of transition metal dichalcogenides has sparked scientific interest due to the strong coupling of lattice and electronic degrees of freedom [1, 2]. It is presumed that semimetals in this class of materials possess unique Weyl fermions in the bulk and Fermi arc surface states. This offers a platform for realizing many exotic physical phenomena [3, 4, 5, 6, 7, 8, 9, 10, 11, 12, 13]. The electronic structure of type‐II topological Weyl semimetals, which break Lorentz symmetry, has been reported for $T_{d}$‐${\mathrm{MoTe}}_{2}$ using angle‐resolved photoemission spectroscopy (ARPES) [10, 11, 14, 15, 16, 17, 18, 19].

**FIGURE 1:**
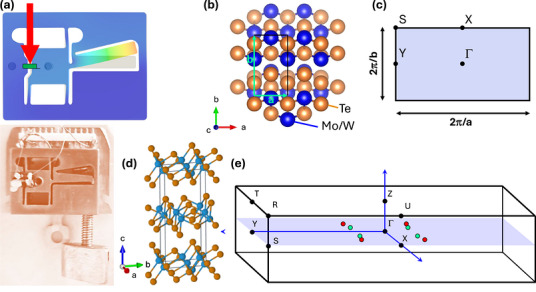
(a) Geometry for the photoemission experiments: The red arrow indicates the direction of the incident X‐ray beam on the sample (green). An exaggerated stage displacement from the starting position (translucent gray overlay) is shown, as predicted by finite element method (FEM). Tensile strain is applied perpendicular to the light scattering plane, i.e., along the (short) a‐axis. The bottom image shows a the strain device on the sample holder with contacts. (b) Top view of the orthorhombic lattice structure of $T_{d}$‐${\mathrm{Mo}}_{0.91}W_{0.09}{\mathrm{Te}}_{2}$. Blue (yellow) balls indicate the Mo/W (Te) atoms. Straight lines mark the unit cell in direct space. (c) The corresponding Γ‐X‐Y section of the Brillouin zone in reciprocal space is shown to illustrate the crystallographic notation and the relative orientations. (d) A three‐dimensional view of the orthorhombic lattice structure of $T_{d}$‐${\mathrm{Mo}}_{0.91}W_{0.09}{\mathrm{Te}}_{2}$. Straight lines mark the unit cell in direct space. (e) The corresponding Brillouin zone in three‐dimensional reciprocal space with the red and green dots indicating the Weyl points.

Normally, the stabilization of the low‐temperature $T_{d}$ phase of ${\mathrm{MoTe}}_{2}$ requires careful temperature control during cooling [20]. However, it has been shown that substituting Mo with W atoms causes a transition from the hexagonal 2H phase to the 1T’ phase at room temperature [21, 22, 23, 24, 25, 26, 27, 28, 29]. Below 170 K, the trigonal 1T’ phase reversibly transforms into the orthorhombic $T_{d}$ phase, see Figure 1b,d.

Theoretical works have predicted that compounds of the ${\mathrm{Mo}}_{x}W_{1-x}{\mathrm{Te}}_{2}$ series are Weyl semimetals [12, 13, 29, 30, 31]. According to ab initio calculations, the momentum‐ and energy‐space distance between a pair of Weyl nodes and the Fermi arc lengths vary depending on the tungsten concentration [29]. However, direct observation of the Fermi arcs remained elusive because they are poorly isolated from the bulk bands [32].

Strain engineering in dichalcogenides is widely used to control the performance of electronic and spintronic devices [33, 34, 35, 36]. Theoretically, it has been shown that the band structure of ${\mathrm{MoTe}}_{2}$ can be reversibly controlled under tensile strain [37], significantly impacting the electronic transport properties. Magnetoresistance increases at low temperatures and high magnetic fields when uniaxial tensile strain is applied along the b‐axis (long axis), and decreases by a similar amount when tensile strain is applied along the a‐axis (short axis). The observed large in‐plane electric anisotropy is coupled with the structural transition from the 1T’ phase to the $T_{d}$ phase, and the phase transition can be tuned with tensile strain. The changes in magnetoresistance suggest the presence of a nontrivial spin‐orbital texture of the electron and hole pockets in the vicinity of Weyl points. Recently, it was predicted that tensile strain along the a‐axis (short axis) is a crucial factor in modulating the bandgap opening in ${\mathrm{MoTe}}_{2}$ [38].

Although experimental studies have demonstrated that uniaxial mechanical strain affects the electronic transport properties of ${\mathrm{MoTe}}_{2}$, there has been no direct experimental evidence of strain‐induced changes to its electronic band structure. This is because combining ARPES with tunable strain devices has remained an experimental challenge [39, 40].

In this study, we demonstrate that mechanical strain is a clean and reversible method of tuning the electronic states of solid‐state materials. To achieve this, we performed hard X‐ray ARPES under uniaxial strain on W‐doped ${\mathrm{MoTe}}_{2}$. Hard X‐ray ARPES extends the probing depth, enabling it to match the properties obtained by transport measurements. Uniaxial tensile strain along the Γ‐Y direction (see Figure 1c) increases the Fermi surface in the same direction. We find that this increase is linear with increasing strain, ε, until a saturation is reached near 0.2%. Below this critical strain, the effect is reversible; above, the changes are irreversible, which indicates plastic deformation or delamination. Additionally, strain reduces the density of states with heavy masses at the Γ point near the Fermi level. This coincides with a decrease in resistivity as the strain increases.

## 2 Results

The two‐dimensional nature of the $T_{d}$‐${\mathrm{Mo}}_{0.91}W_{0.09}{\mathrm{Te}}_{2}$ lattice structure is evident in Figure 1d. The Mo and W atoms form a planar arrangement accompanied by Te atoms that are nearest neighbors, located above and below each Mo/W plane. The interaction between the Mo/W‐Te sheets is dominated by van der Waals bonding, which does not significantly affect the two‐dimensional electronic states.

The definition of the orthorhombic unit cells in direct and reciprocal space is not uniquely used in the literature. We follow the convention that the lattice constants are ordered as a<b<c (see Figure 1b–d).

**FIGURE 2:**
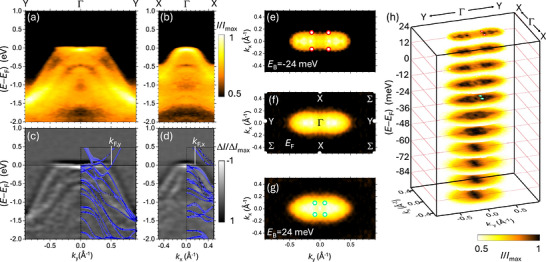
High‐resolution soft X‐ray ARPES of the orthorhombic $T_{d}$‐${\mathrm{Mo}}_{0.91}W_{0.09}{\mathrm{Te}}_{2}$ compound was measured at 25 K. The photon energy is 440 eV corresponding to $k_{z}=24G_{001}$. (a) Section $I(E_{B},k_{x},k_{y}=0)$ of the photoemission intensity along the Γ‐Y direction. (b) Section $I(E_{B},k_{x}=0,k_{y})$ of the photoemission intensity along the Γ‐X direction. (c,d) The same data as in (a,b) but after applying the Laplacian derivative operator to enhance the band position. Theoretically calculated data from Ref. [22] is overlaid. (e–g) Constant energy sections $I(E_{B},k_{x},k_{y})$ at the indicated binding energies, with the positions of the Weyl points sketched in. The indicated Weyl points are not measured but rather reproduced from the theoretical prediction. Photoemission intensity from above the Fermi level stems from thermally excited states. The two types of Weyl points are indicated by red and green circles between the electron and hole pockets. (h) Three‐dimensional representation of the photoemission intensity $I(E_{B},k_{x},k_{y})$. Each constant energy section is normalized by its maximum intensity to enhance the visibility of the electronic states. According to the Fermi distribution function, the absolute photoemission intensity increases with increasing binding energy.

We first performed soft X‐ray ARPES measurements with a higher energy resolution (ΔE=34 meV) than in the hard X‐ray regime (ΔE=130 meV) to compare the experimental band structure to the first‐principles calculations results. Our ARPES spectra shown in Figure 2 agree well with the first‐principles calculations of the occupied electronic states (see Ref. [37]). This allows us to infer the band structure also at energies above the Fermi level, where the Weyl points and Fermi arcs occur.

We present the extracted energy dispersion along the high‐symmetry directions Γ‐X and Γ‐Y in Figure 2a,b. In the section along the Γ‐Y direction (Figure 2a), we observe one electron band and two hole bands at the Fermi level. The electron band appears as increasing energy with increasing momentum $k_{y}$ close to the Fermi level. The energy dispersion along the Γ‐X direction, shown in Figure 2b, shows only the hole band at the Fermi level.

The visibility of the band dispersion is enhanced by the Laplacian derivative operator, as shown in corresponding Figure 2c,d. We compare the energy dispersion with an ab initio calculation [22] of the electronic states for ${\mathrm{Mo}}_{0.91}W_{0.09}{\mathrm{Te}}_{2}$. We find that the calculated band structure agrees well with our experimental data.

Figure 2e–g shows the constant energy sections near the Fermi level. At the Fermi level (see Figure 2f), we observe an ellipsoidal pocket, elongated along the Γ‐Y direction. Its shape is similar to the hole pocket centered at the Γ point and the two electron pockets shifted toward the Y points. These results are consistent with earlier findings considering the energy resolution ΔE=34 meV at a photon energy of 440 eV. As the constant energy section moves from the Fermi level to higher binding energies, the central hole pocket expands while the electron pockets shrink, as shown in Figure 2g. This results in a less elongated ellipsoidal shape.

At a negative binding energy (see Figure 2e), the ellipsoidal shape becomes more elongated and exhibits a richer intensity structure. We observe photoemission intensity above the Fermi level, albeit at a much lower intensity, due to thermal excitation ($4k_{B}T=9$ meV). In this energy range, the exponential decrease of the photoemission intensity resulting from the Fermi distribution function represents a sharp low‐pass energy filter that effectively enhances our energy resolution. With higher energy resolution, we can distinguish the separation between the central, hole‐like Fermi surface with protrusions directed toward the S points and the ellipsoidal, electron‐like pockets centered at $k_{x}=0$ and $k_{y}=0.3$ Å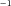, as shown in Figure 2e. Topological Fermi arcs between the Weyl points are expected to occur at the boundary between the electron‐ and hole‐like Fermi surfaces. The indicated positions were adapted from previously reported results. As surface states, they do not appear in our soft X‐ray photoemission spectra.

Figure 2h is a three‐dimensional representation of the measured data array showing the series of constant energy sections up to a binding energy of 100 meV. Each constant energy section has been normalized to its maximum intensity to enhance visibility of the electronic states. The reduction in intensity at higher $k_{y}$ momenta along the Γ‐Y direction with increasing binding energy up to $E_{B}=60$ meV is consistent with the electron‐like band dispersion in this region. The increase in intensity near the Γ point with increasing binding energy is a result of an onset of several hole‐like bands along the Γ‐X direction.

We used HARPES to capture the strain‐induced changes in the bulk band structure. The mean free path of photoemitted electrons is four times greater at 5 keV than at 440 eV, suppressing a significant signal contribution from the topmost layer. This is important because the topmost layer may react differently to strain due to its lack of nearest‐neighbor bonding. Therefore, hard X‐ray photoelectron spectroscopy is better suited for comparison with the transport measurements discussed below.

**FIGURE 3:**
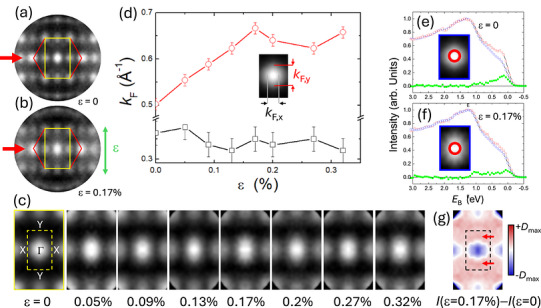
Hard X‐ray ARPES of the orthorhombic $T_{d}$‐${\mathrm{Mo}}_{0.91}W_{0.09}{\mathrm{Te}}_{2}$ compound was measured at 25 K with and without applied strain. The photon energy was 5120 eV, corresponding to $k_{z}=80G_{001}$. (a) The Fermi surface section comprises several unit cells. The yellow rectangle indicates an area of 2×2 Brillouin zones. The red hexagon indicates the parent hexagonal structure of 2H‐${\mathrm{MoTe}}_{2}$. (b) The Fermi surface at the indicated tensile strain along the vertical Γ‐Y direction. (c) A series of Fermi surface maps measured at the indicated strain values. (d) Fermi wave vector along the Γ‐X (black) direction ($k_{F,x}$) and the Γ‐Y (red) direction ($k_{F,y}$) as a function of strain. (e,f) Energy distribution curves (red squares and blue circles) and their difference (green full symbols) integrated over the red and blue outlined areas shown in the inset (both EDCs are normalized to their maximum values to emphasize changes near the Fermi level). (g) Intensity difference map of two Fermi surface cuts, one with strain and one without. The color scale corresponds to a maximum difference of $D_{\max}=0.1$, referenced to the maximum intensity at $E_{B}=1.3$ eV.

The HARPES data recorded at 25 K under external strain are shown in Figure 3. Figure 3a shows a constant energy cut at the Fermi level before applying strain, and Figure 3b with applied tensile strain of ε=0.17% along the vertical, Γ‐Y direction. The Fermi surface section, which has a field of view of 7 Å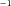, comprises many Brillouin zones. Each bright ellipse indicates a Γ point. The intensity pattern, which is ellipsoidally shaped, corresponds to that observed with soft X‐ray excitation (see Figure 2g), albeit with lower energy resolution. The applied strain results in a change in the intensity distribution near the Γ point as well as an increase in the background intensity (see Figure 3b). Due to the limited energy resolution in the hard X‐ray regime, no further details can be resolved.

We now focus on the central Brillouin zone. The yellow rectangle indicates an area comprising four Brillouin zones that fill the central part of the red hexagon, which highlights the trigonal 1T’ parent structure of the high‐temperature phase.

Figure 3c shows a series of Fermi surface sections measured with increasing tensile strain along the a‐axis. Qualitatively, with increasing strain we observe an increasing elongation of the ellipsoids along the vertical Γ‐Y direction. To quantify this elongation in the horizontal and vertical directions, we analyzed the momentum distribution curves along the X‐Γ‐X and Y‐Γ‐Y paths and determined the Fermi wave vectors along both directions, as indicated in the inset of Figure 3d. The Fermi wave vector $k_{F,x}$ is independent of the applied strain in the direction perpendicular to the applied strain (horizontal axis), but the Fermi wave vector parallel to the applied strain $k_{F,y}$ increases linearly with increasing strain. The increase in $k_{F,y}$ reaches a maximum at ε=0.17% and remains constant with larger strains. We confirmed that the increase in $k_{F,y}$ along the Y‐Γ‐Y direction is completely reversible at ε=0.13%. However, after relaxing the strain from ε=0.34% to zero, the surface exhibits wave‐like deformations. This suggests that a strain greater than ε=0.17% results in a plastic deformation of the sample.

Energy distribution curves in the hard X‐ray regime are an effective measure of the bulk density of states. Figure 3e,f compares energy distribution curves integrated over the entire Brillouin zone (blue circles) and over a smaller region centered at the Γ point (red squares). Without strain, the photoemission intensity near the Γ point shows a steeper increase at the Fermi level compared to the intensity integrated over the entire Brillouin zone. The difference spectrum (green squares) exhibits a sharp maximum at a binding energy of 0.1 eV. This maximum is strongly suppressed in the strained case, indicating a decreased density of states near the Γ point. Without strain the maximum difference between the Γ point and the average value corresponds to an 18% decrease, referenced to the maximum intensity value at $E_{B}=1.3$ eV (see Figure 3e). At a strain of ε=0.17% the corresponding maximum difference is only 10% (see Figure 3f).

Please note that the Fermi distribution function generally decreases the intensity near the Fermi level. This also causes the intensity difference to decrease as the Fermi level is approached. When comparing photoemission intensities and transport properties, it is more meaningful to consider the values at the Fermi level. At the Fermi level ($E_{B}=0$), the integrated photoemission intensity increases from 0.16 to 0.21 as the strain increases to ε=0.17% (the blue circles in Figure 3e,f)). This increase corresponds to a 30% strain‐induced increase in the total density of states. Conversely, the intensity near the Γ point (the red squares in Figure 3e,f) decreases from 0.26 to 0.25, corresponding to a 5% strain‐induced decrease in the density of states. These values indicate that the changes in the density of states are clearly momentum dependent, with spectroscopic weight shifting from the Γ point to states with larger momentum.

To determine the momentum distribution of the difference in the density of states between the unstrained and strained states, we plot the difference in the corresponding constant energy section at $E_{B}=0.1$ eV in Figure 3g. The negative (blue) region near the Γ point is consistent with the decreased intensity observed in the energy distribution curves. Positive values (red), i.e., larger density of states, appear between the Γ and the Y points (indicated by red arrows).

**FIGURE 4:**
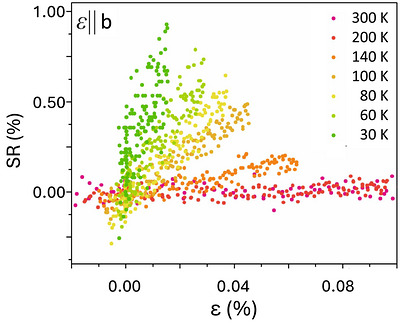
The temperature dependence of the relative resistivity change (SR) is shown as a function of the applied strain ε. Measurements were taken while cooling down to 30 K in a zero magnetic field.

Our HARPES data thus suggest strain‐driven changes in the electronic structure near the Fermi level, which should modulate conductivity. To test this, we performed electrical transport measurements with both electric current and tensile strain applied along the b‐axis. The results of temperature dependent measurements are presented in Figure 4, where the relative change in specific resistivity (ρ) as a function of applied strain, SR=(ρ(ε)−ρ(0))/ρ(0), is presented for several temperatures. In the high‐temperature 1T’ phase (between 200 and 300 K), no change in resistance is observed with applied strain. Below the phase transition temperature from 1T’ to $T_{d}$, strain‐induced resistance changes increase significantly. At the lowest temperature of 30 K, the increase in resistivity amounts to 1% with just 0.01% tensile strain. Here, within error limits, we observe a linear increase in resistivity with increasing strain, without hysteresis.

Our results are qualitatively consistent with transport measurements of pristine $T_{d}$‐${\mathrm{MoTe}}_{2}$ [37], in which an increase in resistivity was observed with increasing strain when the tensile strain was applied parallel to the b‐axis. However, when strain is applied parallel to the a‐axis, increasing strain leads to an increase in conductivity, as observed in Ref. [37].

## 3 Discussion

The structural response of the $T_{d}$ phase of ${\mathrm{MoTe}}_{2}$ to uniaxial strain was previously investigated by density functional theory [37]. The lattice structure under uniaxial tensile strain is determined by the Poisson ratio, $\gamma =\varepsilon_{t}/\varepsilon_{l}$, which describes the transversal strain relative to the longitudinal strain. The reported anisotropic Poisson ratios of ${\mathrm{MoTe}}_{2}$, which we assume to be similar to $T_{d}$‐${\mathrm{Mo}}_{0.91}W_{0.09}{\mathrm{Te}}_{2}$, are $\gamma_{ab}=0.19$ and $\gamma_{ac}=0.96$6 for a tensile strain along the a‐axis and $\gamma_{ba}=0.31$ and $\gamma_{bc}=0.54$ for a tensile strain along the b‐axis [37]. These results indicate a significant anisotropy in the elastic properties, contrasting with earlier models that assumed isotropic magnetoelasticity [41].

**FIGURE 5:**
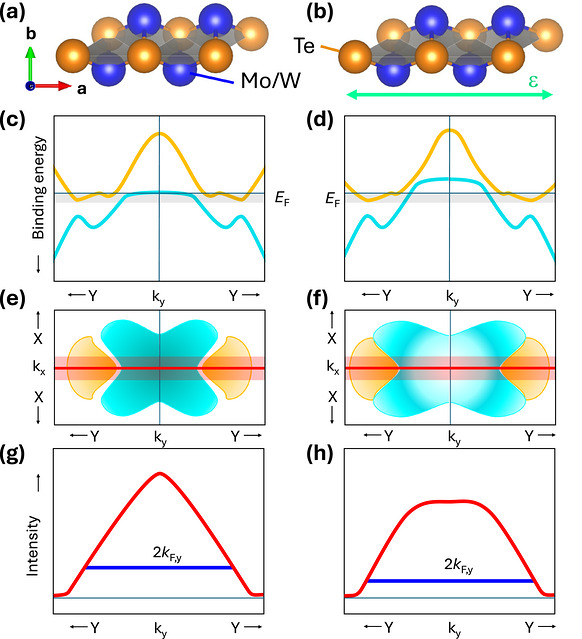
Sketches of the structural and electronic modifications from the pristine case (a,c,e,g) to the case where tensile strain is applied along the (short) a‐axis (b,d,f,h), i.e. along the zigzag lines of the Mo/W atoms. All strain‐related effects are exaggerated to enhance visibility. (a,b) Schematic lattice structure of the unstrained and strained $T_{d}$‐${\mathrm{Mo}}_{0.91}W_{0.09}{\mathrm{Te}}_{2}$ lattice plane. Blue (orange) spheres indicate the Mo/W (Te) atoms. The gray shaded prisms indicate the nearest neighbor environment. (c,d) Band dispersion of the hole‐like and electron‐like bands along the Γ‐Y direction near the Fermi level for the pristine (c) and strained (d) cases. The two bands that generate the topological Weyl points at the avoided crossing points within the gray‐shaded energy range are sketched. (e,f) Sketches of the two‐dimensional local density of states at the Fermi level averaged over the gray energy interval in (c,d) indicating the valence states relevant for electronic transport. Profiles of the density of states are shown in (g,h) along the shaded area in (e,f). These profiles highlight the bell‐shaped profile with $k_{F,y}$ values indicated by the blue bar.

Figure 5a,b illustrates the geometry of the nearest‐neighbor bonding between Mo/W and Te atoms, both without strain and with tensile strain applied along the a‐axis (the strain shown here is exaggerated to emphasize the changes). In the strained state, not only does the distance between two Mo/W atoms and between Mo/W and the nearest neighboring Te atoms increase, but the bonding angles are also modified. The band structures of the unstrained lattice and of the lattice under strain along the a‐axis are shown in Figure 5c,d. The sketches of the band dispersions of the most significant hole‐like (blue) and electron‐like (orange) bands were created from ab‐initio calculated results reported in Ref. [37]. Changes in the band dispersions are observed around the Γ point and along the Γ‐Y direction. The most prominent change is the shift of the hole‐like band near the Γ point to a higher kinetic energy. This shift also results in an upward shift of the electron‐like bands in the avoided‐crossing region, thereby altering the band overlap. Since the electronic states near the avoided‐crossing region inherit topological properties from the topological Weyl‐states existing in these areas, their strain‐induced depletion likely changes electronic transport properties.

The band shift reduces the density of states near the Fermi level. This behavior is illustrated in Figure 5e,f, which depict the momentum distribution of the density of states averaged over a small energy range around the Fermi level relevant for transport properties (marked by gray areas in Figure 5c,d). A profile along the Y‐Γ‐Y direction (see Figure 5g,h) reveals the different distribution of the density of states along this direction. The profile for the unstrained case reveals a Gaussian shape with a sharp maximum (see Figure 5g). In contrast, tensile strain along the a‐axis causes the maximum to flatten and the Fermi wave vector $k_{F,y}$ to increase (see Figure 5h).

These characteristic strain‐induced modifications of the density of states near the Fermi level align well with our experimental observations. Along the Y‐Γ‐Y path, we observe an increase in $k_{F,y}$ of the momentum distribution curves, resulting in an elongation of the elliptical pattern (see Figure 3d). Consequently, more electronic states with larger momentum become occupied.

Thus, an increase in occupied states with large momentum and topological character shifts the weight of states contributing to electronic transport toward states that experience less scattering. Additionally, electronic states near the Γ point that occupy a flat band region and possess heavy mass become unoccupied. The decrease in scattering and the lower average transport‐mass of electrons result in a pronounced increase in electrical conductivity.

In contrast, tensile strain along the b‐axis causes changes in the electronic band structure in the opposite direction [37]. Therefore, one might expect the electrical resistivity to increase in this case. Our experimental results confirm this expected behavior, showing resistivity increases with increasing tensile strain along the b‐axis (see Figure 4). The onset of the strain‐induced increase in resistivity coincides with the crystallographic phase transition from the 1T’ phase to the orthorhombic $T_{d}$ phase. Since topological states comprising Weyl points and their related Fermi arcs have been predicted only in the $T_{d}$ phase and not in the 1T’ phase, it could be speculated that the observed changes in resistivity induced by strain are caused by the occupancy or de‐occupancy of topological states.

## 4 Conclusion

The possible presence of Weyl points and Fermi arcs in $T_{d}$‐${\mathrm{MoTe}}_{2}$ has sparked scientific interest. Since the hybrid Mo 4d‐Te 5p orbitals couple directly to the lattice, applying strain to this material is expected to modify the orbital texture of the electron and hole pockets and affect the inter‐pocket electron scattering. Indeed, experiments have shown changes in the electrical transport properties of $T_{d}$‐${\mathrm{MoTe}}_{2}$, and ab initio calculations have explained these changes [37] by changes in the electronic states. However, direct observation of changes in electronic bands due to uniaxial strain has proven elusive.

To this end, we performed experiments on $T_{d}$‐${\mathrm{Mo}}_{0.91}W_{0.09}{\mathrm{Te}}_{2}$. Substituting Mo with W atoms stabilizes the 1T’ and $T_{d}$ phases, with the phase transition between them being reversible. Furthermore, partial substitution of Mo with W increases the spin–orbit‐induced band gaps and the corresponding Fermi arc lengths. Through angle‐resolved photoemission spectroscopy with soft X‐ray excitation, we confirmed that the bulk electronic states of $T_{d}$‐${\mathrm{Mo}}_{0.91}W_{0.09}{\mathrm{Te}}_{2}$ are similar to those of $T_{d}$‐${\mathrm{MoTe}}_{2}$ and align with density functional theory predictions.

In‐operando angle‐resolved hard X‐ray photoemission spectroscopy revealed distinct changes in the bulk electronic structure with respect to tensile strain. The observed changes for tensile strain along the a‐axis confirm previously reported ab initio calculations, suggesting a depletion of electronic states with heavy masses and high scattering rates, leading to an increase in conductivity. In contrast, tensile strain along the b‐axis results in a significant increase in resistivity.

Our results directly confirm the idea that tensile strain can be used to adjust the electronic properties of two‐dimensional electronic materials. This finding should motivate further theoretical investigations into these phenomena.

## 5 Experimental Section

### 5.1 Sample Fabrication

Single crystals of 1T’‐${\mathrm{Mo}}_{0.91}W_{0.09}{\mathrm{Te}}_{2}$ were grown by iodine vapor transport. High‐purity tungsten and molybdenum wire and tellurium pellets were sealed at one end of evacuated quartz ampules (inner diameter 12 mm, length 200 mm) in a near stoichiometric ratio with approximately 15% excess tellurium (3 mg/${\mathrm{cm}}^{3}$) together with iodine granules (5 mg/${\mathrm{cm}}^{3}$). The ampules were subjected to a temperature gradient of 3 K/cm, with the hotter end containing the starting materials at 900 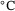. The growth time for crystals forming near the colder end was 900 h.

The low‐temperature orthorhombic structure of $T_{d}$‐${\mathrm{Mo}}_{0.91}W_{0.09}{\mathrm{Te}}_{2}$ was confirmed by X‐ray diffraction at 100 K. Its lattice constants are a=3.469 Å, b=6.311 Å, and c=13.894 Å.

The sizes of crystals were up to 5×5 ${\mathrm{mm}}^{2}$ in area and 0.1 mm thick. The samples are metallic. For the photoemission experiments, we prepared the samples through mechanical exfoliation of the bulk ${\mathrm{Mo}}_{0.91}W_{0.09}{\mathrm{Te}}_{2}$ crystal. To do so, we glued the crystal onto a flag‐style sample plate with conducting epoxy glue. Then, we pressed a Kapton tape onto the bulk crystal and removed the tape with the topmost layers in vacuum. The crystal was then transferred under vacuum onto a He‐cooled (25 K) sample stage on a high‐precision, 6‐axis hexapod manipulator of a time‐of‐flight momentum microscope.

### 5.2 Strain Device

The strain device, as depicted in Figure 1a, uses a screw‐driven, monolithic flexure stage with a right‐angle motion transfer implemented by an L‐shaped lever. The screw is rotated using the same dual‐shaft wobble stick used to load flag‐style sample holders, but equipped with a home‐built single‐degree vernier scale. The screw's axial advance pushes the lever, which redirects the motion by 90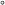 to actuate a double‐parallelogram flexure stage. This linkage provides a 15:1 reduction from screw advance into stage displacement.

The screw tip remains in continuous contact with the lever due to the restoring force of the flexure system, ensuring well defined actuation. The device offers a usable stage displacement range exceeding 10 μm, enabling strains in excess of 1% in our geometry (where strain is applied over a length of 1 mm). Finite Element Method (FEM) was used to map the screw advance to the stage displacement and confirm a linear displacement response with minimal parasitic motion within the intended operating range (see Figure 1a). The displacement‐strain relation depends on the effective gauge length (the gap between the fixed ends of the sample) and can be calibrated using a strain gauge. Calibration has been performed at room temperature. The screw‐driven monolithic structure, unlike piezo based actuation, does not change significantly upon cooling, thus avoiding low‐temperature complications of strain gauges (i.e. a strong resistance increase and brittleness) and piezo actuators (i.e. strongly reduced range). The main source of systematic error in the strain values, ε, is the measurement of the effective gap length, which was estimated to be 0.1ε. Applying strain orthogonal to the incident X‐ray beam reduces the risk of obscuring the beam path (e.g., by the adhesive fillets or wiring) at the 22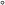 incidence angle. The right angle linkage was chosen to meet the form factor and access constrains inside the time‐of‐flight momentum microscope, while enabling a compact, wire‐EDM fabricable mechanism with sufficient range and precision.

### 5.3 Photoemission

Photoemission experiments in the soft X‐ray range were conducted at beamline P04 of the storage ring PETRA III at DESY in Hamburg, Germany [42]. Using soft X‐ray excitation, we measured the intensity distribution of the direct transitions in four‐dimensional energy‐momentum space $I(E_{B},k_{x},k_{y},k_{z})$, which is the spectral density function modulated by matrix elements that account for the photo excitation probability for a given initial $k_{i}$ and final $k_{f}$ state [43, 44]. For a given photon energy hν and binding energy $E_{B}=E_{F}-E$, the final photoelectron states are located on a spherical shell with a radius (for units Å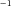 and eV)

(1) $$k_{f}=0.512\sqrt{h\nu -E_{B}+V^{*}}$$

Here the inner potential $V^{*}\approx 10$ eV is referenced to the Fermi energy and the transferred photon momentum leads to a rigid shift of the free‐electron final state sphere by the vector with absolute value k(hν)=2πν/c along the direction of the photon beam [45]. The kinetic energy of the emitted photoelectrons is determined by their time of flight and the Fermi edge is used as a reference for $E_{B}=0$. The photon energy range used in the soft x‐ray experiments is 300 to 500 eV, with energy resolution of 34 meV. The pattern observed on the detector represents the photoelectron intensity distribution as a function of the transverse momentum $k_{\left|\right|}$.

Photoemission experiments on strained samples were conducted at the hard X‐ray beamline P22 of the storage ring PETRA III at DESY in Hamburg, Germany [46, 47, 48]. The footprint of the photon source is approximately 5×20 $\mu m^{2}$. A Si(311) crystal monochromator yielded an energy resolution of 130 meV at a photon energy of 5000 eV.

Angle‐resolved photoelectron spectroscopy in the hard X‐ray range notably increases the inelastic mean free path of the escaping photoelectrons. Accordingly, the HARPES results represent bulk properties rather than surface properties, which is crucial, given that the surface strain potentially differs from that in the bulk.

To address the limitations of the low cross‐section and low signal‐to‐background ratio in the hard X‐ray regime, we used time‐of‐flight momentum microscopy [42]. This technique enables highly efficient acquisition of three‐dimensional photoelectron intensity data sets, $I(E_{B},k_{x},k_{y})$, as a function of binding energy, $E_{B}$, and momentum $k_{x},k_{y}$. Processing the data to reduce noise limits the momentum resolution to 0.1 Å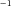 for the results presented below.

Photoemission data were collected for two hours at each strain value. Details of the data evaluation procedure are described in Refs. [45, 48, 49, 50].

### 5.4 Transport Measurements

We used General Electric varnish to glue the sample to the copper sample holder. We then used a four‐probe geometry with silver electrical contacts. We performed electric transport measurements in a temperature range of 1.4 to 300 K and elastoresistance measurements as described in more detail in Ref. [51]. The samples were glued to the top side of a piezoelectric stack (Piezomechanik GmbH), and a miniature strain gauge was glued to the back to monitor the strain variation when voltage was applied to the piezoelectric stack. The crystallographic b‐axis of the ${\mathrm{Mo}}_{0.91}W_{0.09}{\mathrm{Te}}_{2}$ sample was aligned with the poling direction of the piezoelectric stack. Due to the different thermal expansion coefficients of the piezoelectric stack and the sample, the sample experiences a small amount of strain even in the absence of an applied voltage to the piezoelectric stack. However, this effect is small enough that it does not modify the sample's electrical transport properties.

### 5.5 Theory

Alloys of ${\mathrm{Mo}}_{1-x}W_{x}{\mathrm{Te}}_{2}$ were computed within the Coherent Potential Approximation (CPA) [52]. The CPA calculations for the $T_{d}$ structure of ${\mathrm{Mo}}_{0.88}W_{0.12}{\mathrm{Te}}_{2}$ started from the crystal structure determined by XRD at 100 K.

## Conflicts of Interest

The authors declare no conflicts of interest.

## Data Availability

The data that support the findings of this study are available from the corresponding author upon reasonable request.
